# Perception and Modeling of Affective Qualities of Musical Instrument Sounds across Pitch Registers

**DOI:** 10.3389/fpsyg.2017.00153

**Published:** 2017-02-08

**Authors:** Stephen McAdams, Chelsea Douglas, Naresh N. Vempala

**Affiliations:** ^1^Music Research, Schulich School of Music, McGill UniversityMontreal, QC, Canada; ^2^Department of Psychology, Ryerson UniversityToronto, ON, Canada

**Keywords:** musical timbre, emotion, pitch register, musical instruments, valence, tension arousal, energy arousal, preference

## Abstract

Composers often pick specific instruments to convey a given emotional tone in their music, partly due to their expressive possibilities, but also due to their timbres in specific registers and at given dynamic markings. Of interest to both music psychology and music informatics from a computational point of view is the relation between the acoustic properties that give rise to the timbre at a given pitch and the perceived emotional quality of the tone. Musician and nonmusician listeners were presented with 137 tones produced at a fixed dynamic marking (forte) playing tones at pitch class D# across each instrument's entire pitch range and with different playing techniques for standard orchestral instruments drawn from the brass, woodwind, string, and pitched percussion families. They rated each tone on six analogical-categorical scales in terms of emotional valence (positive/negative and pleasant/unpleasant), energy arousal (awake/tired), tension arousal (excited/calm), preference (like/dislike), and familiarity. Linear mixed models revealed interactive effects of musical training, instrument family, and pitch register, with non-linear relations between pitch register and several dependent variables. Twenty-three audio descriptors from the Timbre Toolbox were computed for each sound and analyzed in two ways: linear partial least squares regression (PLSR) and nonlinear artificial neural net modeling. These two analyses converged in terms of the importance of various spectral, temporal, and spectrotemporal audio descriptors in explaining the emotion ratings, but some differences also emerged. Different combinations of audio descriptors make major contributions to the three emotion dimensions, suggesting that they are carried by distinct acoustic properties. Valence is more positive with lower spectral slopes, a greater emergence of strong partials, and an amplitude envelope with a sharper attack and earlier decay. Higher tension arousal is carried by brighter sounds, more spectral variation and more gentle attacks. Greater energy arousal is associated with brighter sounds, with higher spectral centroids and slower decrease of the spectral slope, as well as with greater spectral emergence. The divergences between linear and nonlinear approaches are discussed.

## Introduction

The relationship between music and emotion has become a widely studied topic. Its existence is undeniable and multiple studies have revealed that for most people the predominant motivation for listening to and engaging in music is its emotional impact (Sloboda and O'Neill, 2001; Krumhansl, 2002; Juslin and Laukka, 2004). Although, there is an increasing amount of research on music and emotion, it remains difficult to draw decisive conclusions about how musical factors contribute to emotion in a piece. In addition to global structural factors such as mode, melody, harmony, tempo, and form (cf. Gabrielsson and Lindström, 2010 for a review), it is likely that acoustic factors of a sound can relay affective information as well. In this paper, we examine musical instrument timbre and the audio descriptors derived from the sound signal that contribute to its timbre in relation to a three-dimensional model of perceived affect. We also develop linear regression and nonlinear neural net models to establish a computational link between audio descriptors and perceived emotion ratings, providing a basis for a music informatics approach to the role of timbre in emotion perception.

A three-dimensional model of affect measures emotion as a function of valence, tension arousal, and energy arousal (Schimmack and Grob, 2000). This model likely provides a more complete representation of affect than the two-dimensional model with only valence and arousal because tension arousal and energy arousal have been shown to be two distinct measures of activation that should not be collapsed into a single measure (Schimmack and Reisenzein, 2002). Schubert (1999) completed a series of experiments applying a dimensional model of affect to music research and found the dimensional model to be a valid and reliable measure for research involving music and emotion. Furthermore, the three-dimensional model of affect has recently been applied to multiple perceived emotion and music studies (Ilie and Thompson, 2006; Eerola et al., 2009, 2012).

Emotion perception refers to a listener recognizing an expressed emotion, but does not necessitate the feeling of that emotion (Juslin and Västfjäll, 2008). When examining expressed emotion in an entire piece, researchers have mostly focused on pitch combination and order, as well as tempo, which has led to the understanding that structural factors play an important role in emotion perception in music listening. However, listeners' judgments of perceived emotion are not solely based on these structural elements. By altering factors such as amplitude, pitch register, pitch contour, temporal envelope, and filtering in synthesized tone sequences, over two-thirds of the variance in listener's perceived emotion ratings have been explained by the manipulation of the acoustic cues (Scherer and Oshinsky, 1977). Further research supports the notion that finer acoustic features, such as dynamics, articulation, spectrum, and attack character are also factors listeners consider when making emotion judgments (Juslin and Laukka, 2004; Gabrielsson and Lindström, 2010). The latter three factors are components that contribute to the timbre of a sound (McAdams et al., 1995). Furthermore, performers and composers reportedly use timbre as a means of communicating intended emotion to listeners (Holmes, 2011), and parallels involving timbral dimensions have been drawn between perceived emotion in music and in speech sounds (Juslin and Laukka, 2003).

Timbre is a multidimensional acoustic attribute that is composed of spectral, temporal, and spectrotemporal dimensions (McAdams et al., 1995). The term “timbre” refers to a set of perceptual attributes that listeners use to discriminate different sound sources in addition to pitch, loudness, duration, and spatial position. These attributes also contribute to source identity (McAdams, 1993). Additionally, the timbre of acoustic instruments varies with both pitch register and musical dynamics, i.e., a given instrument played in a low register can have a drastically different timbre when played in a high register, and at a given pitch, a change in dynamics (playing effort) is also accompanied by a change in timbre (Risset and Wessel, 1999; Marozeau et al., 2003; McAdams and Goodchild, forthcoming).

The notion that perceived emotion can be judged by non-structural acoustic features is supported by listeners' ability to make emotional judgments on sound samples of extremely short duration, and therefore, with limited acoustic information. In certain cases, as little as 250 ms of a musical excerpt holds enough information to perceive an emotional tone in a consistent manner across listeners (Peretz et al., 1998; Filipic et al., 2010), and even a single note provides listeners with enough cues to form an emotional judgment (Bigand et al., 2005). Furthermore, musical expertise has no impact on musical recognition and emotional judgments based on minimal acoustic information (Filipic et al., 2010). The ability to recognize emotion in such short stimuli emphasizes the importance of examining how individual acoustic factors, such as timbre, contribute to emotion perception in music.

Timbre has been identified as a musical feature correlated with perceived, discrete emotions. In general, bright sounds are associated with happiness, dull sounds with sadness, sharp sounds with anger, and soft sounds with both fear and tenderness (Juslin and Laukka, 2004). When one group of participants in a study by Huron et al. (2014) was asked to judge acoustic properties of 44 Western instruments and another group to judge those instruments' ability to express sadness, all judgments appeared to be based on participants' familiarity and knowledge of the instruments rather than on listening to their timbres. Using the acoustic property judgments, such as the darkness of sound, from the first group of participants as predictors for the sadness judgments of the second group of participants, Huron et al. concluded that acoustic properties of the instruments, the ability of the instrument to make small pitch movements, to play low pitches, and to play quietly predicted sadness judgments. Furthermore, Hailstone et al. (2009) studied timbre as a main factor contributing to emotion perception in music. Listeners heard melodies that possess a strong emotional intent and labeled them with an emotion category in a forced-choice paradigm. The melodies were played by one of four different instruments. There was a significant interaction between instrument and emotion judgment, suggesting that timbral cues may be more important for communicating some basic emotions than others. However, Hailstone et al.'s experiment only studied four instrument sounds and four synthetic sounds with novel melodies, which could possibly confound the emotional expression of the timbre alone.

Eerola et al. (2012) examined timbre in relation to a two-dimensional affect model of valence and energy arousal. The stimuli consisted of 110 recorded samples of musical instrument sounds. Pitch and duration were kept constant at D#4 (311-Hz fundamental frequency) and 1 s, respectively, across all stimuli, and loudness was equalized. Participants listened to the individually presented stimuli and gave affect and preference ratings. The rating scales included valence (pleasant/unpleasant), energy arousal (awake/tired), tension arousal (tense/relaxed), and preference (like/dislike). The three-dimensional model of affect (Schimmack and Grob, 2000) used to collect ratings was reduced to a two-dimensional model for analysis purposes due to a highly collinear relationship between the energy-arousal and tension-arousal dimensions. Furthermore, the valence and preference ratings in their study had a nearly perfect correlation, *r* = 0.97. They also investigated the acoustic cues contributing to affect ratings of individual musical instrument sounds drawn from woodwind, string, brass, and percussion families and equalized in pitch, duration, and loudness. They selected seven audio descriptors based on a principal component analysis of 26 descriptors from the MIRToolbox (Lartillot and Toiviainen, 2007). Valence ratings were primarily explained by a linear combination of the ratio of high- to low-frequency energy, temporal envelope centroid, and spectral skewness, with positive valence resulting from sustained sounds with more energy in the lower-frequency components. Energy-arousal ratings were more related to the ratio of high- to low-frequency energy, temporal envelope centroid, and attack slope, with energetic sounds having sharper attacks and more dominant high frequency components.

It is important to note here that Eerola et al.'s (2012) bipolar emotional valence scale was labeled from unpleasant to pleasant. This provides a clear methodological difference compared to Bigand et al.'s (2005) study in which emotional valence (positive/negative) and pleasantness were rated on two separate scales. The stimuli used in Bigand et al.'s Experiments 2 and 3 were orchestral excerpts of short duration (1 s), sometimes consisting of one single tone. The difference in definition of the scales may have contributed to a key difference in the findings, because Bigand's group found that the emotional valence dimension was not correlated with pleasantness judgments (*r* = 0.08), suggesting that happy music is not necessarily identified with pleasant emotions or sad music with unpleasant emotions. These findings lead to our hypothesis that perceived valence will not be completely correlated with preference ratings in the current experiment.

Pitch height (or register) is also a factor in the perceived affective quality of music and musical sounds. In an extensive review of the literature on musical cues to emotion, Gabrielsson and Lindström (2010) report somewhat inconsistent findings on the link between affective qualities and pitch height. Across the studies reviewed, higher pitch was variously associated with expressions such as happy, serene, dreamy, graceful, exciting, surprising, potent, angry, fearful, and active, whereas lower pitch was characterized as sad, dignified/solemn, vigorous, exciting, boring, and pleasant. These authors suggest that the apparent contradictions may depend on musical context. In the speech domain, higher pitch is associated with arousing and happy affect and a submissive manner and lower pitch with calming and sad affect and a more threatening manner (Frick, 1985), a principle that appears to carry over into music (Juslin and Laukka, 2003; Huron et al., 2006). Two studies have explicitly manipulated pitch height (in addition to other musical parameters) to determine its effect on emotion perception. Eerola et al. (2013) in an emotion category rating paradigm found that at lower pitch, ratings were higher for “scary” and “sad” and lower for “happy” and “peaceful,” whereas at higher pitch, ratings were higher for “happy,” intermediate for “sad” and “peaceful” and lowest for “scary.” Ilie and Thompson (2006) used the 3D model of emotion and found that higher pitch was rated as more pleasant for music, but as less pleasant for speech, than was lower pitch. In an analysis of several thousand instrumental themes, Huron (2008) found that on average pitch height was slightly lower for minor-key than for major-key themes, indicating that composers intuitively use a pitch height in addition to mode to convey emotional tone.

The following experiment aims to further contribute to research regarding the role of timbre and pitch-register-related differences in timbre in affect perception in music by showing that participants' judgments regarding perceived affect vary systematically with timbral qualities of short instrument sounds across their pitch registers. First we examined affect ratings in relation to broader variables such as pitch register and instrument family with a linear mixed model analysis, thus extending Eerola et al.'s research by including different pitch registers. The role of tessitura in emotion perception is a little-studied but important issue, because orchestration treatises often mention the different qualities of instrument sounds in their different registers (e.g., Adler, 2002). Confining a study to a single pitch places some instruments in their optimal middle register and others in extreme low or high registers, which require greater playing effort and may by consequence affect their emotional qualities. By extending the registers, we also expected to find different patterns in the tension-arousal ratings compared to the energy-arousal ratings, supporting a three-dimensional model of affect, instead of a two-dimensional model. Additionally, we expected to find a difference in the perceived valence ratings compared to the preference ratings, highlighting a difference between perceived measures and felt measures. Finally, we expected a significant interaction between pitch register and instrument family for each of the perceived affect ratings, showing perceived emotion ratings may not be the same for all instruments across pitch registers.

To relate the perceptual results to timbral properties, we then examined the relationship between the perceived affect ratings and specific audio descriptors that compose timbre with two techniques: a linear partial least squares regression (PLSR) approach and a nonlinear artificial neural network model. PLSR is a regression method that uses principal components analysis (PCA) as an integral part and originates from the discipline of chemometrics (Geladi and Kowalski, 1986). However, it has been applied more recently within the field of auditory perception (Rumsey et al., 2005; Kumar et al., 2008; Eerola et al., 2009). PLSR analyzes complex correlational relationships between perceptual measures as dependent variables and arrays of acoustical or psychoacoustical variables (hereafter referred to as audio descriptors) as independent variables. It deals with collinearity among independent variables by capturing what is common among them in the principal components. It thus carries out simultaneous data reduction and maximization of covariance between the descriptors and the predicted data, preserving correlational patterns between them. Supervised feedforward artificial neural networks with back propagation (i.e., multilayer perceptrons; Rumelhart et al., 1986; Haykin, 2008) are useful connectionist models that act as nonlinear regression functions for emotion prediction based on audio descriptors. The architecture of the feedforward networks is simple with one hidden layer providing the necessary level of nonlinearity.

## Methods

### Affect ratings

The experimental design isolated timbre as an independent variable, similar to Experiment 1 presented in Eerola et al. (2012), and allowed us to examine register, attack, and playing technique as factors contributing to timbre. Modifications, such as an added valence measure and increased range of pitch register, facilitated a comparison between emotional valence and preference scales as well as examining how changes in register contribute to changes in timbral components that influence emotion ratings.

#### Participants

Forty participants (24 females) were between 18 and 35 years of age (*M* = 23, *SD* = 4.4). Twenty participants reported formal musical training ranging from 7 to 25 years of practice (*M* = 16, *SD* = 5.3), and 14 reported formal training with multiple instruments. The remaining 20 participants reported no musical training at a collegiate level and no more than 1 year of formal music training during childhood. These two groups will be referred to as musicians and nonmusicians, respectively. The difference in age between the two groups was not significant, *t*_(38)_ = −0.42, *p* = 0.68.

#### Stimuli

One hundred and thirty seven recorded instrument sounds were chosen from the Vienna Symphonic Library (Vienna Symphonic Library GmbH, 2011). The recorded samples consisted of sounds played by orchestral instruments from four instrument families: brass, woodwinds, strings, and pitched percussion. Audio signals were sampled at 44.1 kHz with 16-bit amplitude resolution. The stimuli were edited to have a fixed duration of 500 ms with a raised-cosine ramp applied to fade them out over the final 50 ms. The attack of each sound was unaltered. The brass stimuli varied by an attack parameter, having a weak, normal or strong attack, as labeled in VSL. The percussion stimuli varied by mallet material, using a felt, wood or metal mallet. Pitch class was kept constant at D#, and the dynamic level was *forte*, as labeled in VSL. Samples were chosen from the entire range of the instruments with stimuli ranging from D#1 to D#7 (A4 has a fundamental frequency of 440 Hz). Most instruments cannot successfully play from D#1 to D#7, so stimuli were only taken from appropriate and playable registers for each instrument. Although, some instruments can play outside of that range, there were not enough samples to create useful, balanced groups. Furthermore, various techniques, such as flutter-tonguing for brass and woodwinds and vibrato and pizzicato for strings, were also included. A detailed list of the stimuli is provided in Table S1.

#### Procedure

All participants passed a pure-tone audiometric test using a MAICO MA 39 (MAICO Diagnostic GmbH, Berlin, Germany) audiometer at octave-spaced frequencies from 125 Hz to 8 kHz and were required to have thresholds at or below 20 dB HL in order to proceed to the experiment (Martin and Champlin, 2000; ISO, 2004).

**Figure 1:**
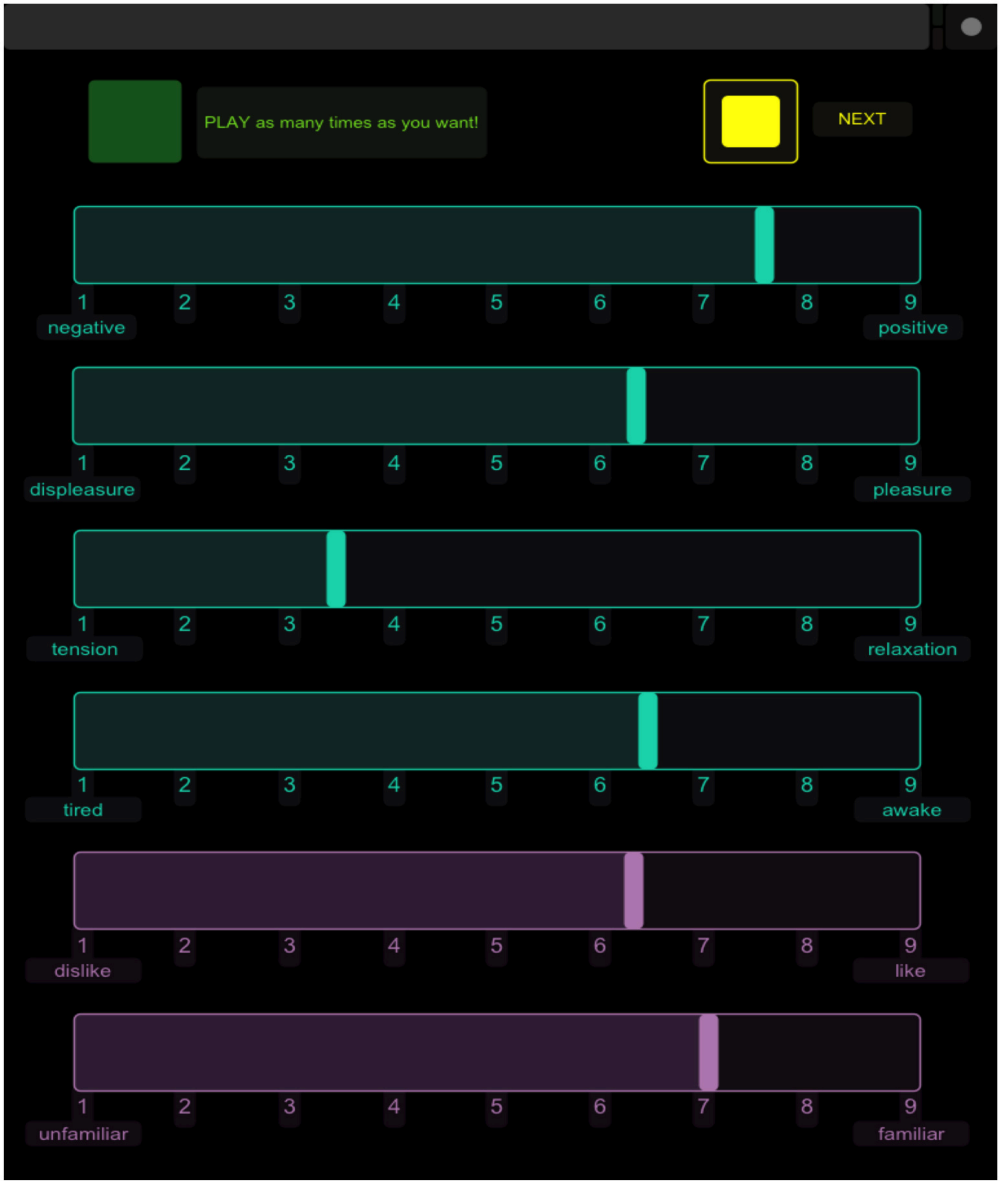
**Screenshot of the experimental interface on the iPad**.

The interface was created in TouchOSC (Hexler.net, 2011) and consisted of a *play* button, six clearly labeled 9-point, analogical-categorical scales (Weber, 1991), and a *next* button. The *next* button was not activated until all six ratings were completed; pressing this button would reset the display to the original position and play the next sound. All 137 stimuli were presented in a randomized order for each participant and each sound could be played as many times as desired, although this information was not recorded. Participants completed six ratings per sound on the 9-point scales. The first four ratings measured perceived emotion and reflected affect dimensions from the three-dimensional model of affect (Schimmack and Grob, 2000) with an additional measure of negative to positive valence. The scales were labeled at the left and right ends with the following pairs: negative/positive (valence), displeasure/pleasure (valence), tired/awake (energy arousal), and tense/relaxed (tension arousal). The participants were also reminded that a rating of 5 would equate to a neutral rating. These four scales were labeled in blue on the iPad interface. The last two ratings measured participants' preference for and familiarity with each sound. These scales were labeled with the pairs dislike/like and unfamiliar/familiar, respectively. These two scales provided a felt rating of personal preference and familiarity and were labeled in purple to differentiate them from the perceived affect ratings. An example of the interface is displayed in Figure 1. Participants were given the following specific instructions: “For the first four scales, you will be rating the degree to which the sound expresses a feeling (NOT how it makes you feel). The last two ratings are how you feel about the sound.” Participants completed the task within an hour and were compensated for their time.

#### Apparatus

Participants completed the experiment individually inside an IAC model 1203 sound-isolation booth (IAC Acoustics, Bronx, NY). The sound samples were played from a Macintosh G5 computer (Apple Computer, Inc., Cupertino, CA), amplified with a Grace Design m904 monitor system (Grace Digital Audio, San Diego, CA), and heard over circumaural Sennheiser HD280 Pro headphones (Sennheiser Electronic GmbH, Wedemark, Germany). The participants were not allowed to adjust the volume. Sound levels were measured with a Brüel and Kjær Type 2205 sound-level meter (A-weighting) connected to a Type 4152 artificial ear (Brüel and Kjær, Nærum, Denmark) to which the headphones were coupled. Stimuli ranged between 59.8 and 77.5 dB SPL (*M* = 65.3, *SD* = 5.4). The participants completed the experiment on an iPad interface (Apple Computer, Inc., Cupertino, CA). The iPad communicated via OpenSoundControl (Center for New Music and Audio Technologies, Berkley, CA) messages over a wireless network with a Max/MSP version 5.1.9 (Cycling '74, San Francisco, CA) patch run on the Macintosh computer. The Max/MSP patch was designed to randomize and play the stimuli as well as to record and output the ratings.

### Control experiment

A control experiment was completed after the original experiment to validate the original interface. The main purpose was to confirm, with a correlation analysis between the control ratings and the original ratings, that no bias resulted from the order and orientation of the rating scales, which remained in a fixed position for every trial and every participant in the original experiment.

#### Participants

Twenty participants (12 females) were between 18 and 42 years of age (*M* = 25, *SD* = 6.5). Ten participants reported formal musical training ranging from 13 to 19 years of practice (*M* = 16, *SD* = 2.5), and seven of those reported formal training with multiple instruments. The remaining 10 participants reported no musical training at a collegiate level and no more than a year of formal music training during childhood. The difference in age between the two groups was not significant, *t*_(18)_ = 0.69, *p* = 0.50.

#### Stimuli

Forty stimuli were selected from the original 137 samples to create a group that was representative of the entire set. Therefore, the control stimuli were brass, woodwind, string, and percussion samples ranging from D#1 to D#7 with weak, normal, and strong attacks. The relevant samples are marked with asterisks in Table S1.

#### Procedure

The instructions and procedure were identical to the original experiment. However, participants were randomly given one of four different interfaces. The interfaces included the same *play* and *next* buttons as the original, but the order of the six scales was changed as well as the orientation (i.e., the end labels) of some of the scales. However, the blue “perceived” scales and the purple “felt” scales were always grouped together to avoid confusion between perceived and felt ratings.

## Results

### Consistency and correlation analyses

We conducted initial reliability analyses and correlations among the scales. All scales had good internal consistency (Cronbach's α for 40 participants = 0.93 for positive/negative, 0.91 for pleasure/displeasure, 0.92 for relaxed/tense, 0.90 for awake/tired, 0.97 for like/dislike, and 0.99 for familiar/unfamiliar). Subsequently, the ratings were averaged across participants for the correlation analysis, so each of the 137 sounds had one measure for each of the six rating scales.

Table 1 displays the correlations between scales within and between the main and control experiments as well as the correlations between the main and control experiments for the 40 sounds common to both studies. Correlations between dependent variables in the main experiment include all 137 sounds, but the correlations within the control experiment and between control and main experiments are only based on the 40 sounds common to both experiments. As all scales in the experiment were very strongly correlated with the designated control, *r*_(38)_ ≥ 0.89, *p* < 0.001, the original interface was confirmed to be valid and reliable. Further analysis is completed on data from the main experiment only.

**Table 1:** **Person's correlation coefficients among ratings of perceived valence, tension arousal, energy arousal, preference, and familiarity for sounds common to both the main and control experiments**.

		**Main**					**Control**			
		**Valen**	**Tens**	**Ener**	**Pref**	**Famil**	**Valen**	**Tens**	**Ener**	**Pref**
Main	Tension	0.46^**^								
	Energy	0.68^**^	−0.29^**^							
	Preference	0.72^**^	0.75^**^	0.19						
	Familiarity	0.56^**^	0.38^**^	0.31^*^	0.66^**^					
Control	Valence	0.89^**^	−0.65^**^	0.44	0.81^**^	0.60^**^				
	Tension	−0.28	0.89^**^	0.47	−0.67^**^	−0.31	0.51^*^			
	Energy	0.56^**^	0.37	0.92^**^	0.01	0.18	0.33	−0.57^**^		
	Preference	0.57^**^	−0.72^**^	0.02	0.89^**^	0.67^**^	0.80^**^	0.69^**^	−0.06	
	Familiarity	0.46	−0.37	0.20	0.57^**^	0.90^**^	0.56^**^	0.31	0.14	0.65^**^

df = 135 for comparisons among Main variables and df = 38 between Main and Control and among Control variables. Bonferroni-corrected

* *p < 0.05*,

** *p < 0.01*.

In the main experiment, the valence scales labeled negative/positive and displeasure/pleasure had a very strong Pearson's correlation of *r*_(135)_ = 0.97, *p* < 0.001. Therefore, in the following analyses, the valence measure will only refer to the negative/positive scale, and the displeasure/pleasure scale will not be analyzed further. The tension-arousal and energy-arousal ratings were only very weakly correlated. There was a strong positive correlation between preference and valence ratings, and a strong positive correlation between preference and tension ratings. However, there was only a very weak positive correlation between preference and energy ratings. Valence was moderately negatively correlated with tension-arousal ratings and strongly correlated with energy-arousal ratings. Familiarity was weakly to moderately correlated with valence, tension arousal, and energy arousal and strongly correlated with preference.

### Linear mixed model analyses

Further statistical analyses employed a linear mixed model method (West et al., 2006), which performs a regression-like analysis while controlling for random variance caused by differences in factors such as participant and stimulus. Because each participant rated all stimuli, the model included crossed random effects for participant and stimulus (Baayen et al., 2008). Specifically, a maximal random effects structure was implemented due to the confirmatory hypothesis nature of the analyses and to reduce Type I errors, i.e., false positives (Barr et al., 2013). Analyses were completed with the R software environment v3.0.2 (www.r-project.org) using the lmer function from the lme4 package (Bates et al., 2014), the Anova function from the Companion to Applied Regression (car) package (Fox and Weisberg, 2011), and the lsmeans package for polynomial contrasts (Lenth, 2013). Welch's unequal variance *t*-test is used to test the significance of the polynomial contrasts.

A linear mixed model analysis was completed for each of the three perceived affect ratings (valence, tension arousal, energy arousal), as well as for the preference and familiarity ratings. Fixed factors examined in these models included instrument family and pitch register of the sounds and musical training of the participants. Attack and playing technique parameters were not included in these initial analyses in order to simplify this model, and because they are not always comparable across instrument families, e.g., flutter tonguing is confined to wind instruments. However, those factors were included in the models for the individual instrument families. Because all participants rated all sounds, a crossed random effects design was implemented and the maximal random effects structure thus included random intercepts for participant with random slopes for family and register, and random intercepts for the stimuli with random slopes for training.

#### Instrument family and pitch register

Type III Wald *F*-test results from the five models are displayed in Table 2. Musical training alone was only a significant predictor of familiarity ratings, although the interaction of training and register was a significant predictor for valence, tension-arousal, and preference ratings. Family was a significant predictor for all ratings except energy-arousal. Register alone and the interaction between register and family both significantly predicted the perceived affect ratings, but not the preference and familiarity ratings. Register was especially influential for energy-arousal ratings. The three-way interaction between training, family, and register was significant for tension-arousal, energy-arousal, and preference ratings.

**Table 2:** **Linear mixed effects model type III wald ***F***-Tests for ratings of perceived valence, tension arousal, energy arousal, preference, and familiarity**.

	***df***	***F***	***p***	***df***	***F***	***p***
	**Valence (*****R***^2^ = **0.53)**			**Tension arousal (*****R***^2^ = **0.43)**		
Intercept	1, 124.4	145.63	< 0.001	1, 121.70	121.96	< 0.001
Training (T)	1, 136.88	0.43	0.512	1, 132.04	1.56	0.214
Family (F)	3, 120.66	7.30	< 0.001	3, 121.11	4.04	0.009
Register (R)	6, 122.01	7.98	< 0.001	6, 120.75	4.15	< 0.001
T × F	3, 104.62	0.24	0.871	3, 95.27	1.38	0.253
T × R	6, 69.38	3.71	0.003	6, 56.34	2.27	0.050
F × R	16, 111.00	2.41	0.004	16, 111.00	2.41	0.004
T × F × R	16, 111.00	1.04	0.417	16, 111.00	2.13	0.011
	**Energy arousal (*****R***^2^ = **0.50)**			**Preference (*****R***^2^ = **0.51)**		
Intercept	1, 124.02	381.45	< 0.001	1, 135.08	120.15	< 0.001
T	1, 137.48	0.05	0.819	1, 96.02	1.97	0.164
F	3, 118.78	1.67	0.178	3, 125.81	10.19	< 0.001
R	6, 112.83	13.27	< 0.001	6, 120.90	1.65	0.139
T × F	3, 91.45	1.44	0.239	3, 94.77	1.10	0.353
T **×** R	6, 53.83	2.10	0.068	6, 58.39	3.89	0.002
F **×** R	16, 111.00	3.09	< 0.001	16, 111.00	1.44	0.135
T **×** F **×** R	16, 111.00	2.30	0.006	16, 111.00	1.99	0.020
**Familiarity (*****R***^2^ = **0.59)**						
Intercept	1, 149.78	112.47	< 0.001			
T	1, 70.46	5.89	0.018			
F	3, 131.80	6.33	< 0.001			
R	5, 120.36	0.82	0.540			
T × F	3, 81.11	2.09	0.108			
T × R	5, 69.51	0.58	0.716			
F × R	16, 111.00	1.85	0.039			
T × F × R	16, 111.00	1.62	0.084			

*N = 5480. All predictors are sum-coded factor variables. The following random effects were included: (a) random intercepts for Participant and Sounds, (b) random slopes for Family and Register (within Participants) and Training (within Sounds)*.

**Figure 2:**
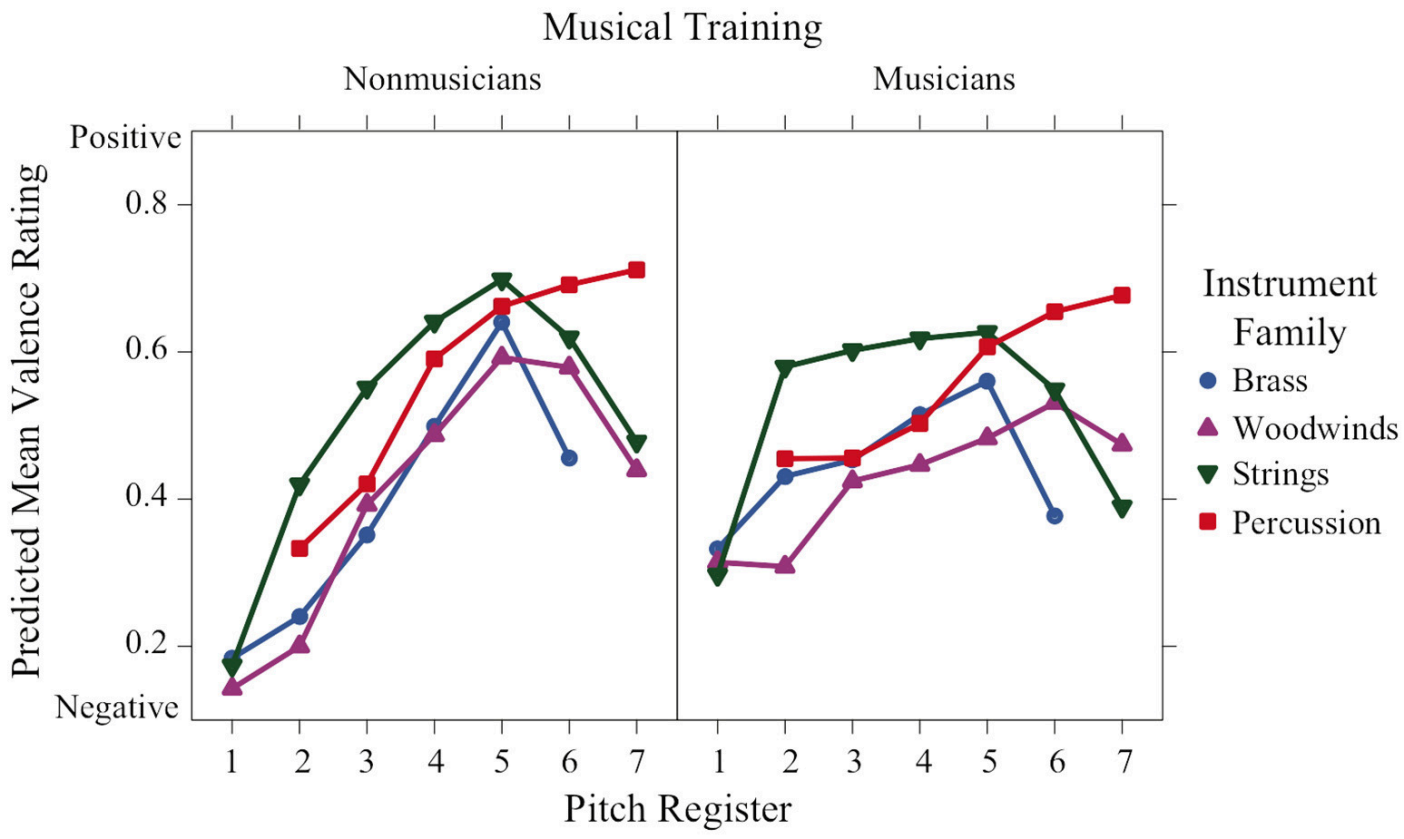
**Means of perceived valence ratings across pitch register for each instrument family and each musical training group**.

**Figure 3:**
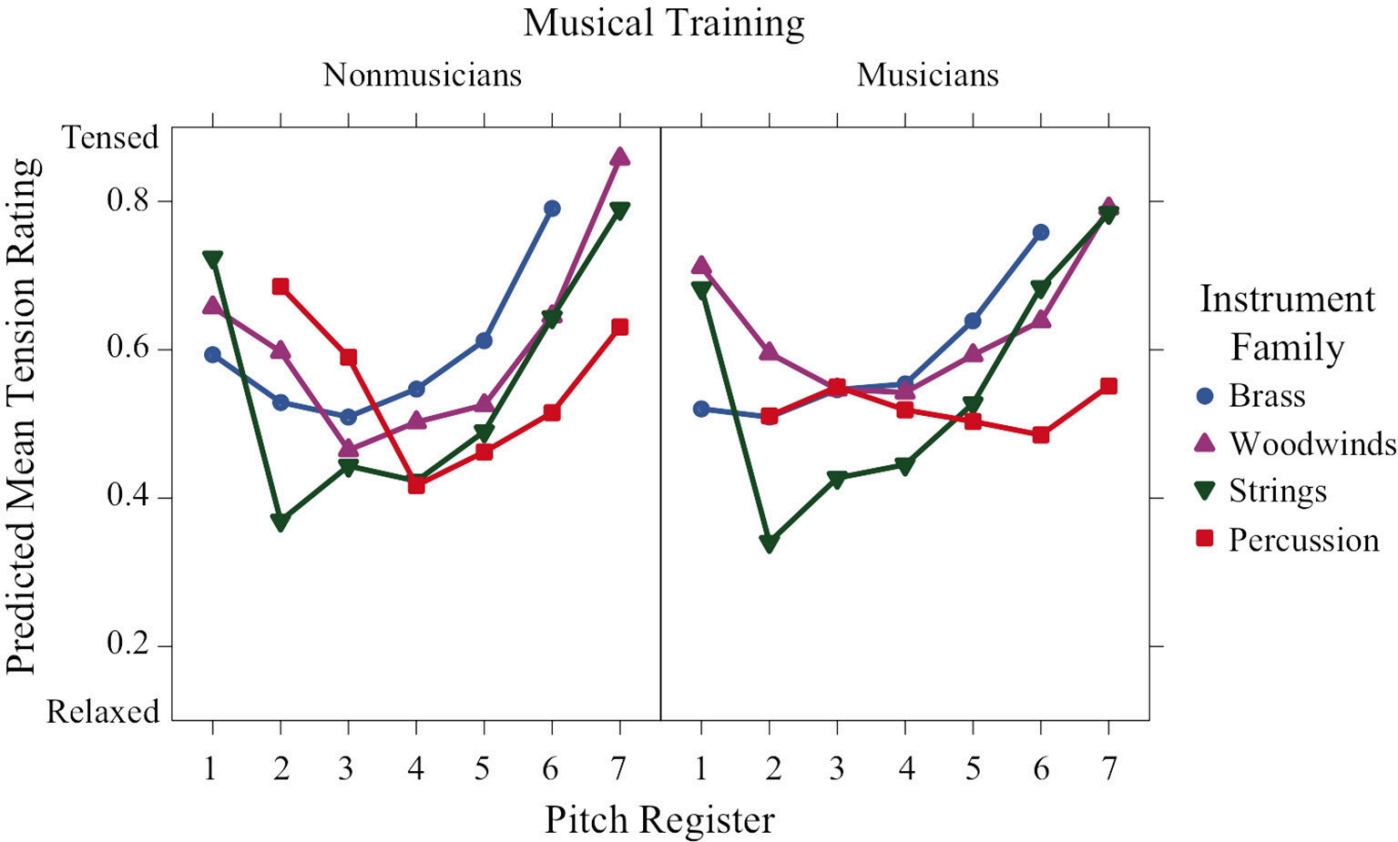
**Predicted means of perceived tension-arousal ratings across pitch register for each instrument family and training group**.

**Figure 4:**
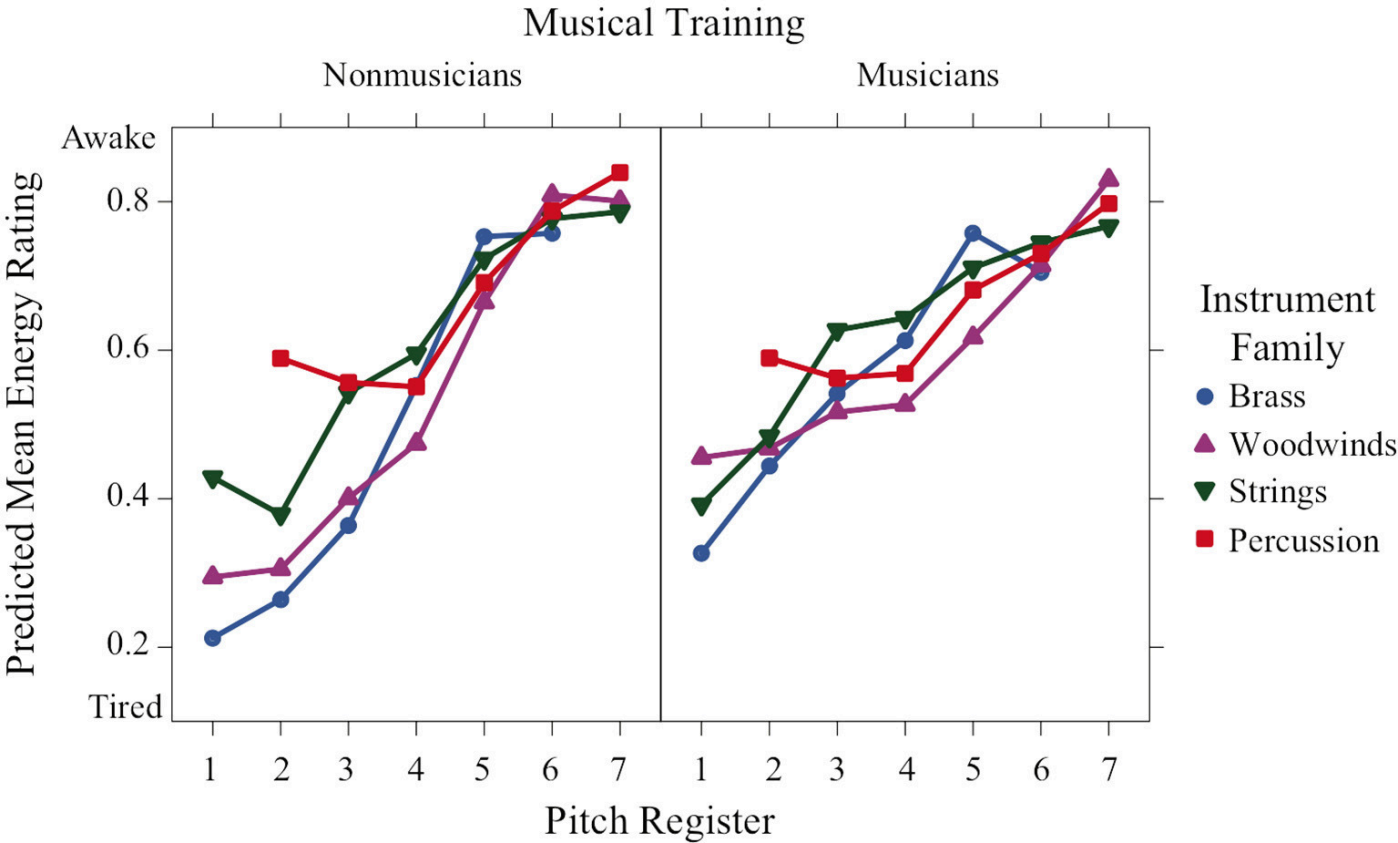
**Predicted means of perceived energy-arousal ratings across pitch register for each instrument family and training group**.

**Figure 5:**
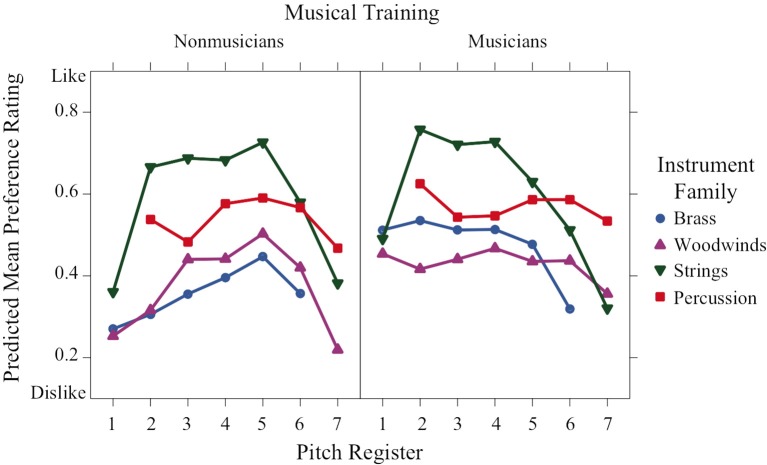
**Predicted means of preference ratings across pitch register for each instrument family and training group**.

**Figure 6:**
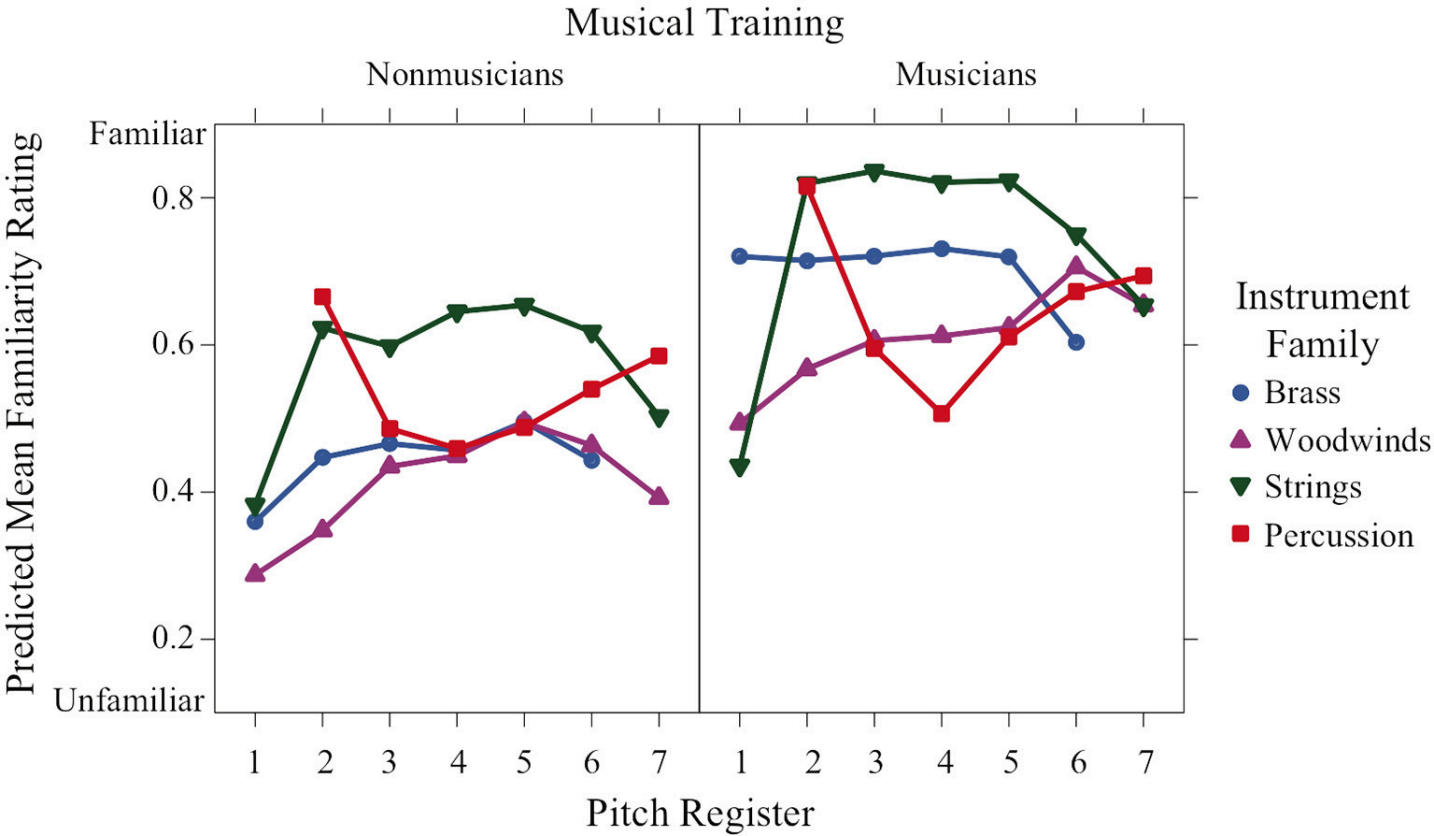
**Predicted means of familiarity ratings across pitch register for each instrument family and training group**.

Figures 2–**6** display plots of predicted means for each rating scale across register for each family and training group. Polynomial contrasts on valence, tension arousal and energy arousal were computed over octaves 2–6 (in which all instrument families are present) with the lsmeans package in R separately for musicians and nonmusicians (see Table S2). For Valence ratings (Figure 2), register was highly significant and globally presents a concave (inverted U-shaped) increasing form with a peak around octave 5 or 6. The polynomial contrasts reveal significant linear increasing and concave quadratic trends for brass, woodwinds, and strings for nonmusicians and for brass and strings for musicians. Woodwinds present an increasing linear trend for musicians as do percussion for both participant groups. The lack of quadratic trend in these latter cases leads to significant interactions between register and family and register and training. There was a main effect of family—strings > percussion > brass > woodwinds. There were valence peaks in the middle-high register with the exceptions of percussion in the higher registers for both groups and woodwinds in the highest octave for musicians as indicated by training × register and family × register interactions.

Tension-arousal ratings (Figure 3) were highly significant for register and followed a convex increasing form, with most families peaking at the lowest and highest octaves. There was a significant training × family × register interaction indicating different patterns across the two groups. The convex increasing trend was apparent for all families in the nonmusician training group, except that the percussion were convex decreasing. Musicians' ratings were similar for brass, woodwinds and strings, but ratings for the percussion family remained relatively neutral across all the registers as indicated by a lack of either linear or quadratic trend.

Register was a highly significant predictor for energy-arousal ratings (Figure 4), and a strong linear trend is visible across registers, with lower registers perceived as more tired and high registers perceived as more awake. Additionally, the register × family interaction was significant. This can be seen, specifically in the percussion family ratings in the second octave (the lowest octave for percussion sounds in this experiment), which were higher than the ratings of the other families in this octave, Welch's unequal variance *t* > 5.02, *p* < 0.0001. Furthermore, the significant training × family × register interaction can be seen when comparing the differences in energy-arousal ratings between the families in the low registers: in the nonmusician group, the families are more spread out in the first three octaves, whereas the ratings of the musician group are more similar across families, even in the low registers.

Similarities among instrument families were slightly less apparent in the graphs of preference and familiarity ratings compared to the perceived affect ratings. Instrument family was a significant predictor of preference ratings (Figure 5), and string sounds in mid-register octaves 2–5 were the most preferred by both musicians and nonmusicians, Welch's *t* ≥ 2.57, *df* ≥ 110.63, Bonferroni-corrected *p* < 0.0021, with three exceptions (strings vs. percussion for nonmusicians in octaves 2 and 4, and for musicians in octave 5). In line with a significant training × family × register interaction, the musicians' preference ratings for brass and woodwind sounds were relatively neutral at lower and mid-register pitches, then decreased in higher registers, whereas nonmusicians preference ratings for brass and woodwind sounds were low for low and high registers, but increased to a neutral rating around octave 5. This pattern in the nonmusicians' ratings is similar to that found in the perceived valence ratings.

Familiarity ratings varied significantly as a function of both training and family (Figure 6). Not surprisingly, they were significantly higher for the musician group than the nonmusician group. There was also a significant family × register interaction depicted by the higher ratings of string sounds in octaves 2–6 compared to the string sounds in octaves 1 and 7. This trend is inversed for percussion sounds, where the highest familiarity ratings occurred in the lowest and highest octaves (2 and 7, respectively).

#### Attack strength and playing technique

To examine attack strength and playing technique as possible factors contributing to affect ratings, the dataset was separated by instrument family, and 20 linear mixed models were created: one for each of the five ratings for each of the four instrument families. All of the models included fixed factors of training and register. Attack strength was included in the brass and percussion models, and technique was included in the brass, woodwind and string models. This separation was necessary because of the lack of different attack strengths in the woodwind and string samples and the lack of different playing techniques for the percussion samples. As with the full models, a maximal random-effects structure was specified for each instrument family model. The brass familiarity, string preference and familiarity, and percussion familiarity models did not converge. Reducing the random effects structure by removing register as a random slope allowed the models to converge, although this removal increases likelihood of Type I errors. That being said, neither attack strength nor technique was a significant predictor for any individual instrument family model.

#### Summary

Valence ratings have a nonlinear concave relation to register, with more positive valence in the middle registers, apart from percussion for which valence progresses from negative to positive from the lowest to the highest register. The effect of register depends on both musical training and instrument family. Nonmusicians gave more negative ratings in the lowest registers compared to musicians. Globally strings have the most positive valence followed by percussion, brass, and woodwinds in order of increasingly negative valence.

Tension-arousal ratings have a nonlinear convex relation to register. Again, the effect of register depends on training and instrument family. Percussion sounds for musicians seem to be unaffected by register and remain at a middle level of tension arousal. Globally, brass receive the highest excitation ratings followed by woodwinds, percussion and strings, which were rated as calmest.

Energy arousal ratings increase monotonically with register, but again they depend on training and instrument family. The ratings are quite similar across families for both groups of participants in octaves 4–7 for nonmusicians and across all octaves for musicians. In the lower octaves, the ratings are spread out for nonmusicians with percussion being rated as most awake followed by strings and then by woodwinds and brass, which are rated similarly.

Preference ratings have a nonlinear concave relation to register that depends on family and training and resemble valence ratings in their form. Familiarity ratings are higher for musicians than nonmusicians. They are concave with respect to register for strings, convex for percussion, and unaffected by register for brass and woodwinds.

### Acoustic descriptors

Due to its multidimensionality, it is necessary to account for multiple acoustic properties when examining timbre (McAdams et al., 1995). There are numerous audio descriptors derivable from the sound signal that can be categorized as spectral, temporal, or spectrotemporal properties of a sound. The following analysis investigates the relationship between the quantitative descriptors and the perceived affect ratings. The tool we use is the Timbre Toolbox (Peeters et al., 2011) as recently updated, corrected, and validated by Kazazis et al. (2016).

The Timbre Toolbox (Peeters et al., 2011) calculates temporal descriptors, such as attack time, spectral descriptors, such as spectral centroid, and spectrotemporal descriptors, such as spectral variation over time, in Matlab (The MathWorks Inc., Natick, MA). There are three stages of computation. First, the input representations of the signal are computed. The Timbre Toolbox has several input representations. The ones we used here included the temporal energy envelope and a Short-Term Fast-Fourier Transform (STFT) with a frequency scale transformed to a physiological scale related to the distribution of frequencies along the basilar membrane in the inner ear as modeled by a scale (ERB-rate) derived from the Equivalent Rectangular Bandwidth (Moore and Glasberg, 1983). To calculate the temporal energy envelope of a given audio signal, the amplitude of the analytic signal, i.e., the signal with no negative-frequency components (Smith, 2007), is given by the Hilbert transform of the audio signal. The amplitude of the analytic signal is then low-pass filtered with a third-order Butterworth filter with a cutoff frequency of 5 Hz, resulting in the temporal energy envelope input representation.

In the second stage of computation, scalar and time-series descriptors are extracted from different input representations. To estimate the attack portion of the signal, the “weakest-effort method” (Peeters, 2004) is applied so that thresholds to detect the start and end time of the attack are not fixed but determined as a proportion of the maximum of the signal's energy envelope. Log-attack time, attack slope, and temporal centroid are calculated from the temporal energy envelope input representation. Log-attack time is the *log*_10_ of the duration (in seconds) of the attack portion of the signal, and attack slope is the averaged temporal slope of the energy envelope during the attack portion of the signal. Additionally, the temporal centroid is a measure of the center of gravity of the energy envelope of the signal.

Each of the spectral descriptors is calculated from the ERB-transformed STFT representation with Hamming time window of 23.2 ms with a hop size of 5.8 ms, thereby giving a time series for each descriptor. As described by Peeters et al. (2011), spectral centroid is a measure of the center of mass of the spectrum and is perceptually related to the “brightness” of the sound. Spectral spread refers to the standard deviation of the spectrum around the spectral mean value and spectral skewness refers to the degree of asymmetry of the spectrum around the mean. Spectral kurtosis examines the flatness of the distribution around the mean value of the spectrum and can indicate a flat, normal, or peaky distribution. Spectral slope is a linear regression over the spectral amplitude values. Spectral decrease is the average of the set of spectral slopes between the fundamental frequency and the frequency of the *k*th harmonic. Spectral rolloff refers to the frequency below which 95% of the signal energy is contained. Spectral variation is a measure of the change in the spectral shape over time, quantified as one minus the normalized correlation between the spectra of successive time frames. Spectral flatness captures the noisiness of the signal and varies between completely “tonal” in the sense of being composed of clear, isolated frequency components and completely noisy. Spectral crest measures the degree of emergence of the most intense frequency component above the average amplitudes of the whole spectrum.

Finally, the third stage of computation considers the median and interquartile range (IQR) values of time-series descriptors to represent both central tendency and variability, respectively (Peeters et al., 2011). Time-series descriptors include spectral centroid, spread, skewness, kurtosis, slope, decrease, rolloff, variation, flatness, and crest. Adding the three temporal descriptors gives the 23 descriptors listed in Table 3.

**Table 3:** **Definition of acoustic descriptors from the timbre toolbox**.

	**Acoustic descriptor**	**Definition**	**Derivative values**
Spectral	Centroid (log)	Center of gravity of the spectrum	Med^*^, IQR^*^
	Spread	Standard deviation of the spectrum around the mean	Med, IQR^*^
	Skewness	Asymmetry of the spectrum around the mean	Med^*^, IQR^*^
	Kurtosis	Flatness of spectrum around the mean	Med, IQR
	Slope	Linear regression over the spectral amplitude values	Med, IQR
	Decrease	Average of slopes between F0 and 2nd to *k*th harmonic	Med^*^, IQR^*^
	Rolloff	Frequency below which 95% of the signal energy is contained	Med, IQR^*^
	Variation	Variation of the spectrum over time	Med^*^, IQR^*^
	Flatness	Ratio of the geometric and arithmetic means of the spectrum	Med^*^, IQR^*^
	Crest	Ratio of the spectral maximum to the arithmetic spectral mean	Med^*^, IQR^*^
Temporal	Attack time (log)	Duration of the attack portion of the sound	^*^
	Attack slope	Rate of change of energy over time in the attack portion	^*^
	Centroid	Center of gravity of the energy envelope	^*^

For time-varying spectral descriptors, both the median (Med) and interquartile ranges (IQR) are computed over the duration of the sound, so each of these descriptors produces two measures.

* *Indicates the 17 descriptors included in partial least-squares regression and neural network analyses (see text)*.

### Partial least-squares regression

We completed a PLSR to examine the relation of the audio descriptors to the set of affect ratings. PLSR couples multiple linear regression with principle components analysis. Furthermore, we applied a five-fold cross-validation model to each PLSR in which the *n* cases are divided into five subsets, and the model is trained on four subsets and then predicts the remaining subset. The subsets are then rotated so that the training and prediction steps are applied to all combinations of the subsets. In addition to calculating *R*^2^ as an evaluation of the model fitness, cross-validation also allows for the calculation of predictive relevance *Q*^2^, the squared cross-validation prediction error summed across the five-folds (Wold et al., 2001).

We used the 23 Timbre Toolbox descriptors described above. The median values of spectral time series provide spectral information, and IQR measures as spectrotemporal information represent the variability of the descriptor over time. The PLSR and a subsequent correlation analysis were both completed in Matlab (The MathWorks Inc., Natick, MA).

**Figure 7:**
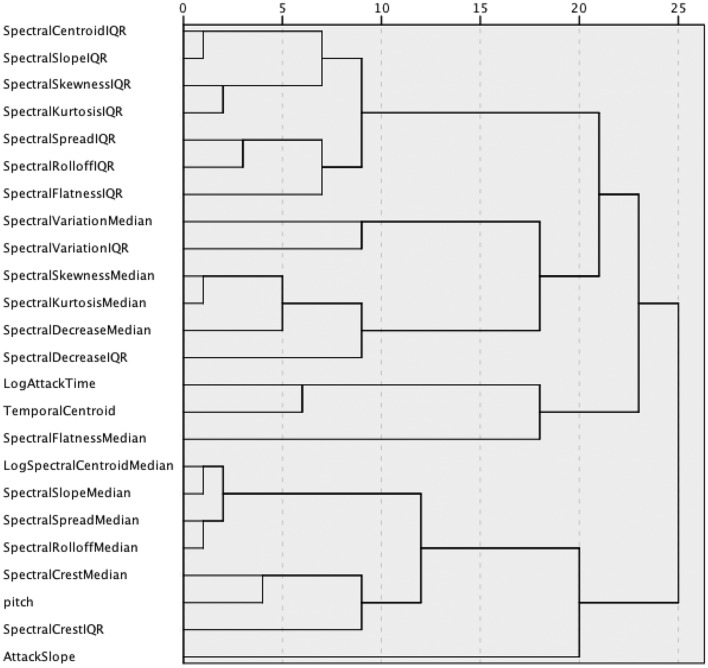
**Dendrogram of the hierarchical cluster analysis of descriptor values across the entire stimulus set**.

An initial analysis of collinearity among descriptors across the complete sound set was performed. The 137 values for each descriptor were correlated with those of every other descriptor, and a hierarchical cluster analysis with average linkage was performed on the correlation matrix. The resulting dendrogram is shown in Figure 7. Several pairs of descriptors join at very low levels indicating high collinearity. A PCA was conducted on the whole set of descriptor values for the 137 sounds. The PCA resulted in errors when including all 23 timbral descriptors plus the nominal pitch of the sounds, because the correlation matrix was not positive definite. Based on the hierarchical cluster analysis, six descriptors that were highly correlated with others were removed: Spectral Slope median and IQR, Spectral Spread median, Spectral Rolloff median, Spectral Kurtosis median, and IQR. These descriptors are highly correlated (*r* > 0.905) with Spectral Centroid median for Spectral Slope, Spread, and Rolloff medians, with Spectral Centroid IQR for Spectral Slope IQR, and with Spectral Skewness median and IQR for Spectral Kurtosis median and IQR, respectively. When these six were removed, the PCA gave a strong Kaiser-Mayer-Olkin index of 0.691, and the removal didn't much affect the total variance explained by the PCA (reduction by 2.2% of the variance explained) or its dimensionality (five components in both cases). Removing pitch as a factor reduced the explained variance by <1%, so it was not included in subsequent analyses either.

The PLSR was thus completed with the group of 17 measures (independent variables) shown with asterisks in Table 3. It was conducted for each of three dependent variables: mean ratings across participants of valence, tension arousal, and energy arousal for each of the 137 stimuli. Based on a threshold eigenvalue of 1, the procedure selected three principal components (PC) for valence and energy arousal, and four components for tension arousal. The upper and lower benchmarks of the model are measured by *R*^2^ (explanatory power) and *Q*^2^ (predictive power). We also computed the root mean squared error (MSE) between the mean ratings and the PLSR estimates. These values are displayed in Table 4. The valence and energy arousal ratings are better modeled than the tension arousal ratings; although all three models have low RMSE. The loadings of each descriptor on each PC are listed in Table 5. They can be interpreted as vector coordinates of the 17 predictors in the three- or four-dimensional spaces of the PCs or as their contributions to each PC.

**Table 4:** *****R***^**2**^, ***Q***^**2**^, and RMSE results for the PLSR models predicting perceived valence, tension arousal, and energy arousal**.

	**Valence**	**Tension arousal**	**Energy arousal**
*R*^2^	0.6617	0.5605	0.7535
*Q*^2^	0.6032	0.4406	0.6867
RMSE	0.0827	0.0841	0.0772

**Table 5:** **Loadings of each audio descriptor on the Principal Components (PC) for each emotion dimension in the PLSR analysis**.

	**Valence**			**Tension Arousal**				**Energy Arousal**		
	**PC1**	**PC2**	**PC3**	**PC1**	**PC2**	**PC3**	**PC4**	**PC1**	**PC2**	**PC3**
Spec centroid Med	6.18	**-8.53**	1.55	**10.39**	−3.95	1.81	−0.73	**9.21**	−5.05	0.89
Spec centroid IQR	3.25	−4.45	5.35	2.43	−1.67	−2.07	2.83	3.51	−6.72	3.90
Spec spread IQR	1.46	−2.92	4.27	−0.43	1.38	−2.36	−0.30	0.97	−3.63	5.55
Spec skewness Med	−5.88	**8.74**	−1.89	−**10.29**	3.98	−1.07	0.89	−**8.91**	5.92	−0.64
Spec skewness IQR	0.67	−0.41	4.96	−2.29	0.41	−2.28	3.76	−0.49	−3.50	5.20
Spec decrease Med	−**8.10**	4.88	−1.12	−**8.04**	5.88	−3.77	1.60	−**10.03**	0.86	−0.73
Spec decrease IQR	−4.38	3.96	−1.83	−6.82	4.41	−2.93	−**6.32**	−6.21	3.24	−1.61
Spec rolloff IQR	1.20	−1.39	4.61	−1.59	0.50	−2.34	2.26	0.30	−3.21	5.87
Spec variation Med	−2.18	−4.58	−**6.63**	0.99	**8.79**	4.41	−3.74	−1.40	2.32	2.38
Spec variation IQR	−4.81	−5.62	−3.20	1.68	**9.33**	−0.30	−2.94	−3.53	−1.65	2.26
Spec flatness Med	−2.10	−6.56	2.56	4.67	0.23	−5.97	−1.65	−0.07	−**9.26**	−1.43
Spec flatness IQR	1.87	−3.27	3.87	0.92	1.77	−1.54	−2.20	1.87	−1.55	**6.58**
Spec crest Med	**8.20**	−0.22	−0.05	3.89	−5.53	4.97	−3.79	**8.65**	5.09	0.75
Spec crest IQR	6.81	−1.46	1.50	2.38	−3.54	1.85	−5.53	6.87	2.47	1.77
Log attack time	−7.47	−3.19	6.05	4.49	4.06	−7.34	2.65	−4.85	−3.15	**6.56**
Attack slope	7.99	3.02	−5.67	−4.36	−3.72	**7.71**	−3.36	5.35	3.93	−5.19
Temporal centroid	−**8.38**	−1.59	2.56	2.65	3.56	−6.34	4.28	−6.29	−3.41	1.54
Partial *R*^2^	0.58	0.06	0.03	0.39	0.09	0.05	0.03	0.72	0.02	0.02

*Loadings greater that 8.0 (or the highest value for a given PC if none are above 8) are shown in bold to highlight the primary contributors discussed in the text*.

#### Valence

Increases in PC1 (58% of the variance explained) are primarily associated with increasing spectral centroid and spectral crest medians, decreasing spectral decrease median, increasing spectral crest variability (IQR), increasing attack slope, and decreasing log attack time and temporal centroid. PC2 (6%) loadings show a positive effect of spectral skewness median and negative effects of spectral centroid and spectral flatness medians. PC3 (3%) is more positive with decreasing spectral variation median and with decreasing log attack time.

#### Tension arousal

PC1 (39%) increases with increasing spectral centroid median and decreasing spectral skewness and spectral decrease medians, with additional negative contribution of spectral decrease IQR. PC2 (9%) increases with increasing spectral variation median and IQR. PC3 (5%) increases with increasing attack slope and with decreasing log attack time and temporal centroid. PC4 (3%) increases with decreasing spectral decrease IQR.

#### Energy arousal

PC1 (72%) increases with increasing spectral centroid and crest medians, with decreasing spectral skewness and spectral decrease medians, with increasing spectral crest IQR, and with decreasing spectral decrease IQR and temporal centroid. PC2 (2%) increases with decreasing spectral flatness median and decreasing spectral slope IQR. PC3 (2%) increases with increasing variability in spectral flatness and increasing log attack time.

Globally, medians of all the time-varying spectral parameters play a stronger role than do measures of their variability or the temporal parameters, although the latter two groups make a significant contribution. The acoustic underpinnings of emotion portrayal by musical instrument sounds thus seem to result from a complex interplay of spectral, temporal, and spectrotemporal factors.

### Neural network model

Previous research has shown that non-linear methods can be particularly useful in situations where linear methods are insufficient to model the relationship between dependent and independent variables. Artificial neural networks may be used as a non-linear regression method (Coutinho and Cangelosi, 2011; Russo et al., 2013; Vempala and Russo, 2013) to predict valence, tension arousal, and energy arousal ratings using timbre descriptors.

We used supervised feedforward networks with back propagation (i.e., multilayer perceptrons) for this purpose (Bishop, 1996; Haykin, 2008; Rumelhart et al., 1986). We built three types of prediction networks—one for valence, one for tension arousal, and one for energy arousal in Matlab. Each type of network consisted of one input layer with 17 units corresponding to the descriptors with asterisks in Table 3, one hidden layer with 3 units, and one output unit corresponding to the mean valence, tension arousal or energy arousal value of participants for that stimulus. Separate networks were trained with valence, tension arousal, or energy arousal as the output unit.

Our training paradigm involved five-fold cross-validation to avoid over fitting the network to any specific partitioning of the training and test sets. To enable cross-validation, we partitioned 135 of the 137 stimuli in our dataset into five equal sets of 27. For each fold, we tested the network on the 27 stimuli within that fold along with the remaining two unused stimuli, after training the network on the four additional folds (i.e., 108 stimuli; see Table S1).

As is common in neural net modeling, all input descriptors were range-normalized in the interval [0, 1] to allow the network to maximize performance by capturing similarities and differences within and across descriptors for all examples of the training set. Connection weights from the input layer to the hidden layer and from the hidden layer to the output unit were initialized to random values between –0.05 and 0.05, allowing for optimal adjustment of hidden units during training. Outputs at the hidden layer were computed using sigmoid functions. The sigmoid or logistic sigmoid function is commonly used in multilayer perceptrons because it has desirable properties. It transforms the data nonlinearly while limiting the range of values between 0 and 1, thus acting as a useful squashing function. For each training stimulus, the squared error between the network's predicted output and the mean participant rating was computed. Changes to connection weights over successive epochs were computed using back propagation of errors with gradient descent and were then stored. After completion of each epoch (i.e., 108 training stimuli), connection weights were updated with the sum of the stored weight changes.

While having more hidden units helps the network converge earlier, and reduces the MSE, it also results in the network becoming overfitted to the training set, thus reducing the network's generalizability. Hence, after initial simulations starting with 7 hidden units, we progressively reduced the number of hidden units down to 3 units, upon noticing that the network's performance was still high for all five-folds of cross-validation. Each network was tested on the set of 29 stimuli. We computed the RMSE as a measure of performance.

The valence and energy arousal networks were trained for 700 epochs, but the tension network took longer to converge, requiring 1000 epochs. All networks successfully converged to a MSE of <0.008. Cross-validation performance for each type of output unit is reported for each of the five-folds along with the mean performance in Table 6.

**Table 6:** **Performance of neural networks modeling valence, tension arousal, and energy arousal**.

**Network**	**RMSE**		
	**Valence**	**Tension arousal**	**Energy arousal**
1	0.0800	0.0814	0.0703
2	0.0801	0.0813	0.0672
3	0.0809	0.0803	0.0672
4	0.0816	0.0853	0.0687
5	0.0825	0.0822	0.0680
Mean	0.0810	0.0821	0.0683

To get a sense of which of the timbre predictors were important for each dependent variable, we used a method developed by Milne (1995). This method computes the size and sign of each feature's contribution to the output by taking into account the connection weights from that feature to the hidden layer, and from the hidden layer to the output unit. We computed feature contributions and averaged them across the five networks used for cross-validation. The mean contribution proportions of the top six timbre features for perceived valence, tension arousal, and energy arousal are reported as percentages in Table 7, along with their signs. A negative sign indicates that increases in the value of the feature are associated with decreases in the emotion dimension. Different combinations of spectral, temporal, and spectrotemporal descriptors contribute to the neural network modeling of valence, tension arousal, and energy arousal. Although, at some level all audio descriptors make some contribution, the primary contributors differ across the rating scales, suggesting acoustic independence among them.

**Table 7:** **Primary timbre descriptor contributions to each type of neural net output unit**.

**Feature**	**Valence**	**Tension arousal**	**Energy arousal**
Spectral centroid median	−	+8.6	+8.3
Spectral spread IQR	−	−7.0	−
Spectral decrease median	−9.5	−	−9.6
Spectral rolloff IQR	−	+6.7	−
Spectral variation median	−10.6	+15.9	−
Spectral variation IQR	−11.0	−	−12.1
Spectral flatness median	−	−7.2	−
Spectral crest median	+10.9	−	+7.7
Spectral crest IQR	−	−	+15.2
Attack slope	+7.9	−7.1	−
Temporal centroid	−15.8	−	−13.9

*The values indicate the mean % contribution and the sign of the relation between the model prediction and the audio descriptor*.

## Discussion

We explored the perceived emotional qualities of 137 isolated tones played by standard western orchestral instruments across their entire pitch ranges and using different playing techniques. One novel aspect of this study on the role of timbre in perceived emotion is that both instrument family and pitch register were varied. It is important to recognize that register affects the timbre of notes produced by each instrument in the sense that several spectral measures are strongly or very strongly correlated with pitch octave [in decreasing order: spectral crest, *r*_(135)_ = 0.826; spectral decrease, *r*_(135)_ = −0.816; spectral centroid, *r*_(135)_ = 0.690; and spectral skewness, *r*_(135)_ = −0.637]. Therefore, timbre varies with register, but not directly as a function of fundamental frequency. The aim was to determine the acoustic properties related to timbre that contribute to ratings by musician and nonmusician listeners on continuous scales of the emotional qualities valence (on both positive vs. negative and pleasure vs. displeasure scales), tension arousal and energy arousal. Listeners also rated preference for and familiarity with each sound. We first discuss the rating data as a function of pitch register, instrument family, and the musical training of the listeners. We then discuss the two approaches to modeling the data with linear PLSR and nonlinear neural nets.

### Listener ratings (ground truth)

The two valence scales were very strongly correlated [*r*_(135)_ = 0.97], and so subsequent analyses were limited to the positive/negative scale. It is worth recalling that using musical excerpts of varying, but not systematically controlled instrumentation, Bigand et al. (2005) found no correlation between pleasantness and positive-negative valence. So other musical properties may distinguish these two dimensions of musical experience.

Linear mixed effects models that take into account variation due to participants and stimulus items revealed strong interactions of the factors pitch register, instrument family, and musical training for all rating scales. Valence and preference had nonlinear concave relations to register indicating that maximally positive valence and preference corresponded to middle registers. The exception to this pattern for valence was the percussion family, which had a monotonic increasing relation to register. The families in order from negative to positive valence were woodwinds, brass, percussion, and strings. Tension arousal had a nonlinear convex relation to register except for the percussion family for musicians, which had a medium tension level across registers. The families in order of decreasing tension were brass, woodwinds, percussion, and strings. Energy arousal had a monotonic relation to register, with only small differences among the families in the lower registers for nonmusicians. Familiarity ratings were higher for musicians than nonmusicians and were concave with respect to register for strings, convex for percussion and unaffected by register for brass and woodwinds. These results can be compared to those of Eerola et al. (2013) who manipulated several musical parameters on musical phrases, including timbral brightness (flute, horn, trumpet in order of increasing spectral centroid) and pitch height (from F3 to B5 in 6-semitone steps, which corresponds to the middle 2.5 octaves of our 6-octave range). These authors found linear contributions of both timbre and register to ratings of “scary” (increasing with brightness, decreasing with register), “sad” (decreasing with brightness and register), and “peaceful” (decreasing with brightness, increasing with register), but not of “happy.” They also found slight quadratic contributions of register to ratings of “scary” (convex) and “peaceful” (concave), but not “happy” or “sad.” It is difficult to compare directly these two sets of results, one being in a dimensional framework and the other in a categorical framework, but they both emphasize the complex mapping of emotion onto these musical parameters. One does note, however, that a stronger nonlinearity appears with a wider range of pitch heights.

Musical training interacted with register and instrument family for all three emotion dimension ratings, and preference ratings as well. It only interacted with family for familiarity ratings. Regarding the valence ratings, musicians tended to perceive low-register sounds as less negative than nonmusicians. For tension arousal ratings, nonmusicians had convex curves as a function of register for all families, whereas for musicians only woodwinds and strings had this form; percussion were unaffected by register, and tension increased monotonically with register for brass. Energy arousal ratings were globally less affected by instrument family and musical training, with the notable exception of results in lower registers where differences in perceived energy arousal between families were found for nonmusicians. As expected, familiarity ratings were higher in the musician group and varied across instrument family. Musicians are more familiar with sounds in extreme registers than nonmusicians, and this familiarity could potentially play a role in the perceived affect ratings. These differences between musical training groups differ from the finding of Filipic et al. (2010) who found no such difference with short musical clips.

In our experiment, there was a moderately strong positive correlation between the perceived valence ratings and preference, but the negative correlation between perceived tension-arousal ratings and preference was slightly stronger. Although, participants typically preferred more positive, less tense timbres, this finding demonstrates that there is not a clear one-to-one relationship between positive valence or tension and listener's preference. Furthermore, pitch register significantly influenced both perceived valence and tension-arousal ratings so that mid-register sounds were rated as more positive and more relaxed than sounds of an extreme high or low register.

We confirmed that listeners can consistently rate the perceived affect of individual sounds from different musical instruments across their pitch registers with short sounds (500 ms). These results are in accordance with those of Eerola et al.'s (2012) Experiment 1 and other studies utilizing short musical samples (Peretz et al., 1998; Bigand et al., 2005; Filipic et al., 2010) in which the participants were able to rate perceived affect in 1-s or 500-ms instrumental music samples with great consistency.

There were a few key differences between our results and those of Ilie and Thompson (2006), on the one hand, and those from Eerola et al.'s (2012) Experiment 1 on the other. First, the tension-arousal and energy-arousal ratings were only weakly, although significantly, correlated in the present study and Ilie and Thompson's, whereas they were strongly correlated in Eerola et al.'s study. As the energy-arousal dimension had a monotonic relation to register, listeners seem to have used primarily spectral cues when making energy ratings and incorporated additional acoustic information when making tension ratings (see discussion of audio descriptors below). Furthermore, valence and preference ratings in this experiment were moderately correlated, whereas they were strongly correlated in Eerola et al.'s study. Listeners did not necessarily prefer sounds with the highest perceived valence. We therefore concur with Ilie and Thompson in emphasizing the importance of differentiating these measures in a larger stimulus context. One crucial difference is the use of a single pitch in Eerola et al. compared to the whole range of registers for each instrument in the present study. This would mean that the sounds from some instruments in their study would be in their extreme high or low registers. Pitch octave in the present study was strongly correlated with valence, *r*_(135)_ = 0.624, *p* < 0.0001, weakly correlated with tension arousal, *r*_(135)_ = 0.242, *p* = 0.004, and very strongly correlated with energy arousal, *r*_(135)_ = 0.849, *p* < 0.0001. So the differential effect of pitch register on the two arousal scales seems to further distinguish them in the current study. Furthermore, the linear mixed model analysis showed that the energy-arousal ratings were strongly influenced by pitch register, and unlike the tension-arousal ratings, were not significantly influenced by instrument family. This finding is a significant contribution to affect and timbre research because it shows that the two arousal dimensions are distinctly perceivable in timbre and not interchangeable, as they are influenced by different factors.

### Linear and nonlinear modeling of the acoustic basis for perceived emotion dimensions

To analyze the contribution to the emotion ratings of acoustic properties related to timbre, we examined 23 acoustic signal parameters taken from the Timbre Toolbox (Peeters et al., 2011), spanning spectral, temporal, and spectrotemporal audio descriptors. Initial hierarchical clustering and principal components analyses suggested reducing these to 17 descriptors due to high collinearity. It is interesting to note that the pitch height descriptor did not make a significant contribution as it was highly collinear with several spectral descriptors, underlining again the fact that timbre and pitch covary strongly in many acoustic musical instruments. A PLSR with these 17 descriptors as predictors of each of the three emotion dimensions allowed us to reduce the dimensionality to three principal components for valence and energy arousal and to four principal components for tension arousal. Additionally, a nonlinear neural network multilevel perceptron model (NN) was programmed with 17 inputs represented by the audio descriptors, three hidden units, and a single output separately modeling the three mean emotion dimension ratings. In both cases, a five-fold cross-validation method was used to estimate the reliability of the models. Several measures compare ground truth values (means across participants for a given emotional dimension) to predicted values (Table 8). Model fitness (*R*^2^) is computed on items in the training set (the 4 groups excluding the test set, see Table S1) for each fold and then averaged across the five-folds. The model's predictive power (*Q*^2^) is computed on the five training sets collectively taken across the five-folds. The prediction error (RMSE) is computed on both training and test sets for each fold and is then averaged across the five-folds. The percent improvement of the NN model over the PLSR model is shown in Table 8, in which a positive sign indicates higher values for NN. One notes that a much better fit is obtained for all three emotion dimensions with the NN models than with the PLSR models (32–78% improvement), as they are all near perfect prediction. The predictive power across training sets is more equivalent for the two techniques, but it still shows more than 10% improvement for the two arousal dimensions with the NN model. The prediction error is roughly equivalent in the two models for valence and tension arousal, and although better for energy arousal with the PLSR model, the NN model still shows 12% improvement (lower error) for this emotion dimension. The nonlinear approach would thus seem to have modest modeling advantages over the linear approach.

**Table 8:** **Comparison of model fitness, predictive power, and prediction error for PLSR and neural network models**.

**Method**	***R***^**2**^			***Q***^**2**^			**RMSE**		
	**Valence**	**Tension**	**Energy**	**Valence**	**Tension**	**Energy**	**Valence**	**Tension**	**Energy**
PLSR	0.6617	0.5605	0.7535	0.6032	0.4406	0.6867	0.0827	0.0841	0.0772
NN-MLP	0.9971	0.9963	0.9983	0.6117	0.4870	0.7658	0.0810	0.0821	0.0683
Percentage of improvement (%)	51	78	32	1	11	12	−2	−2	−12

Table 9 presents the primary audio descriptors that contribute to each emotion dimension model for each technique. The ranks of the six descriptors providing the highest loadings for PLSR or highest percent contribution for NN are shown. Descriptors that make major contributions to both models are highlighted with darker colors. Note firstly that different combinations of audio descriptors make major contributions to the three emotion dimensions, suggesting that they are carried by distinct acoustic properties. Valence is primarily carried by spectral and temporal properties. It is more positive with lower spectral slopes (more high-frequency energy), a greater emergence of strong partials, and an amplitude envelope with a sharper attack and earlier decay. To the contrary, Eerola et al. (2012) found more positive valence ratings with sustained sounds having more low-frequency energy, and Ilie and Thompson (2006) found more positive valence for lower-register sounds. Tension arousal ratings are driven by all three types of descriptors. Higher tension is carried by brighter sounds, more spectral variation and more gentle attacks. This result is coherent with Ilie and Thompson's finding that increase pitch height is associated with increase tension arousal. Energy arousal seems primarily spectral in nature, and greater energy is associated with brighter sounds with higher spectral centroids and slower decrease of the spectral slope, as well as with a greater degree of spectral emergence. The spectral aspect echoes Eerola et al.'s result showing this emotion dimension to be associated with more dominant high-frequency components, although those authors also found sharper attacks to be associated with higher ratings of energy arousal. Ilie and Thompson found no effect of pitch height (and concomitantly, spectral distribution) on energy arousal. The factors that distinguish these three studies is that ours covers a much wider range of pitches, thus augmenting the role played by pitch height and its concomitant timbral attributes related to spectral properties.

**Table 9:** **Ranks of primary audio descriptors contributing to PLSR and NN models**.

**Audio Descriptor**	**Type**	**Valence**		**Tension Arousal**		**Energy Arousal**	
		**PLSR**	**NN**	**PLSR**	**NN**	**PLSR**	**NN**
Spectral centroid median	Spectral	2	–	1	2	3	5
Spectral centroid IQR	Spectrotemporal	–	–	–	–	6	–
Spectral spread IQR	Spectrotemporal	–	–	–	5	–	–
Spectral skewness median	Spectral	1	–	2	–	4	–
Spectral skewness IQR	Spectrotemporal	–	–	–	–	–	–
Spectral decrease median	Spectral	5	5	5	—	1	4
Spectral decrease IQR	Spectrotemporal	–	–	–	–	–	–
Spectral rollof IQR	Spectrotemporal	–	–	–	6	–	–
Spectral variation median	Spectrotemporal	–	4	4	1	–	–
Spectral variation IQR	Spectrotemporal	–	2	3	–	–	3
Spectral flatness median	Spectral	–	–	–	3	2	–
Spectral flatness IQR	Spectrotemporal	–	–	–	–	–	–
Spectral crest median	Spectral	4	3	–	–	5	6
Spectral crest IQR	Spectrotemporal	–	–	–	–	–	1
Log attack time	Temporal	–	–	–	–	–	–
Attack slope	Temporal	6	6	6	4	–	–
Temporal centroid	Temporal	3	1	–	–	–	2

*PLSR is in green and NN in orange. Descriptors that make major contributions to both models are highlighted with darker colors*.

## Conclusion

This study examined timbre and its complex covariation with pitch as musical elements capable of conveying emotion information. It highlights the fact that changes in pitch are accompanied by significant changes in timbral properties as quantified by timbral audio descriptors. It also demonstrates the fact that different intrinsic emotional qualities of musical instrument sounds are carried by different, but overlapping, sets of acoustic dimensions, suggesting that it is their complex combination that specifies emotional tone. This work provides a foundation for work on the acoustic underpinnings of perceived emotion in musical sound that could stimulate additional work in music informatics by providing tools for including timbre in content-based approaches to automatic identification of mood in music (Kim et al., 2010). Future research should apply these results to increasingly ecological studies to validate the relationship between timbre, pitch, and perceived affect in a musical context and examine how that relationship interacts with additional relationships between perceived affect and other musical variables such as dynamics, tempo, harmony and texture. But even on its own, this work provides a rough map of how sounds produced by musical instruments in given registers relate to perceived emotional tone, suggesting basic acoustic characteristics upon which composers capitalize in sculpting musical experience.

## Ethics statement

All subjects gave written informed consent in accordance with the Declaration of Helsinki. The protocol was certified for ethics compliance by the McGill University Research Ethics Board II.

## Author contributions

SM and CD conceived the experiments and interpreted the analyses of behavioral data. CD conducted the experiments and performed the analyses of behavioral data. SM, CD, and NV conducted acoustics analyses and modeling and contributed to the writing of the paper.

## Funding

This work was supported by a grant from the Natural Sciences and Engineering Research Council of Canada (RGPIN-2015-05028) and a Canada Research Chair (950-223484) awarded to SM.

### Conflict of interest statement

The authors declare that the research was conducted in the absence of any commercial or financial relationships that could be construed as a potential conflict of interest.
