# Supplementary material for: Perception and Modeling of Affective Qualities of Musical Instrument Sounds across Pitch Registers
Source: Front Psychol. 2017 Feb 8;8:153. doi: 10.3389/fpsyg.2017.00153 (PMC5296353; doi:10.3389/fpsyg.2017.00153)
Supplement: Supplementary file 1 [file Table1.DOCX]

Supplementary Material

Perception and Modeling of Affective Qualities of Musical Instrument Sounds Across Pitch Registers

Stephen McAdams*, Chelsea Douglas, Naresh N. Vempala

*** Correspondence:** Stephen McAdams: stephen.mcadams@mcgill.ca

**Table S1. Instruments And Octaves From Which Notes Were Drawn, Families, Special Playing Techniques Included In The Stimulus Set, And Groups Used For Cross-Validation Analyses**

| **Instrument Family** | **Instrument** | **Octaves** | **Attack** | **Technique** | **Group** |
| --- | --- | --- | --- | --- | --- |
| **Brass** | Contrabass Tuba | 1, 2 | Strong |  | 1, 2 |
|  | Contrabass Tuba | 1, 2 | Normal |  | 3, 4 |
|  | Contrabass Tuba | 1*, 2 | Weak |  | 5, 1 |
|  | Contrabass Tuba | 1, 2 | Normal | Flutter tongue | 2, 3 |
|  | Tuba | 3, 4 | Strong |  | 4, 5 |
|  | Tuba | 3, 4 | Normal |  | 1, 2 |
|  | Tuba | 3, 4 | Weak |  | 3, 4 |
|  | Tuba | 3, 4* | Normal | Flutter tongue | 5, 1 |
|  | Contrabass Trombone | 1, 2 | Strong |  | 4, 5 |
|  | Contrabass Trombone | 1, 2 | Normal |  | 5, 1 |
|  | Contrabass Trombone | 2* | Normal | Flutter tongue | 2 |
|  | Bass Trombone | 3* | Strong |  | 2 |
|  | Bass Trombone | 3 | Normal |  | 3 |
|  | Bass Trombone | 3 | Normal | Flutter tongue | 4 |
|  | Tenor Trombone | 4 | Strong |  | 3 |
|  | Tenor Trombone | 4 | Normal |  | 2 |
|  | Tenor Trombone | 3, 4* | Weak |  | 1, 3 |
|  | Tenor Trombone | 4 | Normal | Flutter tongue | 5 |
|  | Horn | 3, 4, 5* | Strong |  | 4, 1, 2 |
|  | Horn | 3, 4, 5 | Normal |  | 3, 4, 5 |
|  | Horn | 3*, 4, 5 | Weak |  | 2, 3, 4 |
|  | Horn | 3, 4 | Normal | Flutter tongue | 5, 1 |
|  | Trumpet | 4 | Strong |  | 2 |
|  | Trumpet | 4* | Normal |  | 3 |
|  | Trumpet | 4 | Weak |  | 5 |
|  | Trumpet | 4 | Normal | Flutter tongue | 4 |
|  | Piccolo Trumpet | 5, 6* | Strong |  | 1, 2 |
|  | Piccolo Trumpet | 5, 6 | Normal |  | 3, 4 |
|  | Piccolo Trumpet | 5, 6 | Weak |  | 5, 1 |
|  | Piccolo Trumpet | 5* | Normal | Flutter tongue | 3 |
| **Woodwinds** | Contrabassoon | 1*, 2 | Normal |  | 5, 4 |
|  | Bassoon | 3, 4 | Normal |  | 1, 2 |
|  | Bassoon | 2*, 3, 4 | Normal | Flutter tongue | 3, 4, 5 |
|  | Bass Clarinet | 2*, 3 | Normal |  | 1, 2 |
|  | Bb Clarinet | 4, 5, 6* | Normal |  | 1, 5, 3 |
|  | Bb Clarinet | 3*, 4, 5 | Normal | Flutter tongue | 3, 2, 1 |
|  | Oboe | 4*, 5 | Normal |  | 4, 2 |
|  | Oboe | 4, 5 | Normal | Flutter tongue | 4, 5 |
|  | English Horn | 4, 5* | Normal |  | 1, 2 |
|  | English Horn | 4, 5 | Normal | Flutter tongue | 3, 4 |
|  | Alto Flute | 4 | Normal |  | 5 |
|  | Alto Flute | 4* | Normal | Flutter tongue | 1 |
|  | Flute | 5*, 6 | Normal |  | 3, 2 |
|  | Flute | 5, 6 | Normal | Flutter tongue | 4, 5 |
|  | Piccolo | 7* | Normal |  | 3 |
| **Strings** | Harp | 1*, 2, 3*,4, 5, 6, 7* | Plucked |  | 1, 2, 3, 4, 5, 1, 2 |
|  | Contrabass | 2* | Bowed |  | None |
|  | Contrabass | 2* | Plucked | Vibrato | 4 |
|  | Cello | 3, 4 | Bowed |  | 2, 5 |
|  | Cello | 3, 4* | Bowed | Vibrato | 4, 3 |
|  | Cello | 3*, 4 | Plucked | Vibrato | 5, 1 |
|  | Viola | 5 | Bowed |  | 2 |
|  | Viola | 5* | Bowed | Vibrato | 1 |
|  | Viola | 5 | Plucked | Vibrato | 3 |
|  | Violin | 6*, 7 | Bowed |  | None, 4 |
|  | Violin | 6, 7* | Bowed | Vibrato | 5, 1 |
|  | Violin | 6 | Plucked | Vibrato | 3 |
| **Percussion** | Timpani | 2*, 3 | Metal |  | 5, 2 |
|  | Timpani | 2, 3 | Wood |  | 4, 1 |
|  | Timpani | 2, 3* | Felt |  | 2, 3 |
|  | Gong | 3, 4*, 5 | Felt |  | 4, 5, 1 |
|  | Gong | 3, 4, 5* | Wood |  | 3, 2, 4 |
|  | Gong | 3, 4, 5 | Metal |  | 5, 3, 2 |
|  | Gong | 3, 4 | Bowed |  | 1, 4 |
|  | Celesta | 3*, 4, 5 | Wood |  | 5, 1, 2 |
|  | Celesta | 6*, 7 | Wood |  | 3, 4 |
|  | Glockenspiel | 6, 7* | Metal |  | 5, 1 |
|  | Glockenspiel | 6, 7 | Wood |  | 2, 3 |
|  | Xylophone | 5, 6* | Wood |  | 5, 4 |
|  | Vibraphone | 4, 5*, 6 | Metal |  | 2, 3, 1 |
|  | Crotales | 6, 7* | Metal |  | 4, 5 |

Note: * = 40 sounds selected for the control experiment. Group numbers correspond in order to Octaves in the same line.

**Table S2. Polynomial Contrasts for the Three Emotion Ratings on Different Instrument Families by the Two Participant Groups**

|  |  | **Valence** | | **Tension Arousal** | | **Energy Arousal** | |
| --- | --- | --- | --- | --- | --- | --- | --- |
| **Family** | **Contrast** | **NonMus** | **Mus** | **NonMus** | **Mus** | **NonMus** | **Mus** |
| Brass | Lin | B = .72 *t* = 9.86 *p*< **.0001** | B = .0006 *t*= .008  *p*= .99 | B = .63 *t*= 7.56 *p*< **.0001** | B = .59 *t*= 7.15 *p*< **.0001** | B = 1.37 *t*= 18.70 *p*< **.0001** | B = .74 *t*= 10.04 *p*< **.0001** |
|  | Quad | B = –.60 *t*= –7.74 *p*< **.0001** | B = –.43 *t*=  –5.52 *p*< **.0001** | B = .42 *t*= 4.84 *p*<**.0001** | B = .59 *t*= 7.15 *p*<**.0001** | B = –.18 *t*= –2.30 *p*= .022 | B = –.23 *t*= –2.91 p *= .0036* |
| Woodwinds | Lin | B = .89 *t*= 10.79  *p*<**.0001** | B = .47 *t*= 5.77  *p*<**.0001** | B = .26 *t*= 2.82 *p = .0048* | B = .21 *t*= 2.24 *p*= .025 | B = 1.27 *t*= 15.28 *p*<**.0001** | B = .65 *t*= 7.86 *p*< **.0001** |
|  | Quad | B = –.47 *t*= –5.34 *p*< **.0001** | B = –.15 *t*=  –1.71 *p*= .087 | B = .60 *t*= 5.97 *p*< **.0001** | B = .32 *t*= 3.20 *p*=**.0014** | B = .21 *t*= 2.37 *p*= .018 | B = .23 *t*= 2.65 *p = .0081* |
| Strings | Lin | B = .42 *t*=  5.48 *p*< **.0001** | B = –.17 *t*=  –2.24 *p*= .025 | B = .72 *t*= 8.19 *p*< **.0001** | B = .87 *t*= 9.93 *p*< **.0001** | B = .98 *t*= 12.61 *p*< **.0001** | B = .62 *t*= 8.00 *p*< **.0001** |
|  | Quad | B = –.57 *t*=  –6.23 *p*< **.0001** | B = –.34 *t*= –3.74  *p*=**.0002** | B = .32 *t*= 3.56 *p*=**.0004** | B = .29 *t*= 2.78 p *= .0055* | B = –.14 *t*= –1.46 *p*= .14 | B = –.15 *t*= –1.60 *p*= .11 |
| Percussion | Lin | B = .94 *t*= 13.69 *p*< **.0001** | B = .57 *t*= 7.98 *p*< **.0001** | B = –.37 *t*= –4.65 *p*< **.0001** | B = –.04 *t*= –.56 *p*= .57 | B = .57 *t*= 7.98 *p*< **.0001** | B = .45 *t*= 6.33 *p*< **.0001** |
|  | Quad | B = –.20 *t*= –2.42 *p*= .015 | B = .17 *t*= 2.06 *p*= .040 | B = .61 *t*= 6.53 *p*< **.0001** | B = –.05 *t*= –.50 *p*= .62 | B = .44 *t*= 5.39 *p*<**.0001** | B = .31 *t*= 3.77 *p*= **.0002** |

Note: *df* = 5120. Bonferroni-corrected significant p-values in bold (*p* ≤ .0021), marginal p-values in italics (*p* ≤ .0167) for 24 tests.
